# The 2011 Survey on Hypertensive Disorders of Pregnancy (HDP) in China: Prevalence, Risk Factors, Complications, Pregnancy and Perinatal Outcomes

**DOI:** 10.1371/journal.pone.0100180

**Published:** 2014-06-17

**Authors:** Chun Ye, Yan Ruan, Liying Zou, Guanghui Li, Changdong Li, Yi Chen, Chaoxia Jia, Ian L. Megson, Jun Wei, Weiyuan Zhang

**Affiliations:** 1 Department of Obstetrics, Beijing Obstetrics and Gynecology Hospital, Capital Medical University, Beijing, China; 2 Department of public healthcare information administrative section, Beijing Obstetrics and Gynecology Hospital, Capital Medical University, Beijing, China; 3 Department of Diabetes & Cardiovascular Science, University of the Highlands & Islands, Inverness, United Kingdom; Indiana University School of Medicine, United States of America

## Abstract

Hypertensive disorders of pregnancy (HDP) are a group of medical complications in pregnancy and also a risk factor for severe pregnancy outcomes, but it lacks a large-scale epidemiological investigation in recent years. This survey represents a multicenter cross-sectional retrospective study to estimate the prevalence and analyze the risk factors for HDP among the pregnant women who had referred for delivery between January 1st 2011 and December 31st 2011 in China Mainland. A total of 112,386 pregnant women were investigated from 38 secondary and tertiary specialized or general hospitals randomly selected across the country, of which 5,869 had HDP, accounting for 5.22% of all pregnancies. There were significant differences in the prevalence of HDP between geographical regions, in which the North China showed the highest (7.44%) and Central China showed the lowest (1.23%). Of six subtypes of HDP, severe preeclampsia accounted for 39.96%, gestational hypertension for 31.40%, mild preeclampsia for 15.13%, chronic hypertension in pregnancy for 6.00%, preeclampsia superimposed on chronic hypertension for 3.68% and eclampsia for 0.89%. A number of risk factors for HDP were identified, including twin pregnancy, age of >35 years, overweight and obesity, primipara, history of hypertension as well as family history of hypertension and diabetes. The prevalence of pre-term birth, placental abruption and postpartum hemorrhage were significantly higher in women with HDP than those without HDP. The possible risk factors confirmed in this study may be useful for the development of early diagnosis and appropriate treatment of HDP.

## Introduction

Hypertensive disorders of pregnancy (HDP) are a group of common medical complications in pregnancy, and also a major cause of maternal and neonatal mortality and morbidity [1]. The prevalence of HDP is 8–10% of all pregnancies in the population worldwide [2]. It can develop during pregnancy or delivery, and its clinical presentation is characterized by hypertension, proteinuria and edema. HDP can also trigger some severe forms of maternal complications, such as cardiovascular and cerebrovascular diseases, liver and kidney failure, placental abruption, disseminated intravascular coagulation (DIC) and HELLP syndrome. Under these circumstances, the placenta dysfunction may occur, leading to fetal growth restriction, fetal distress, preterm birth, intrauterine fetal demise, stillbirth and neonatal asphyxia. While HDP has been described as a maternal complications over the last several decades [3], its true etiology and pathophysiology remains unknown; HDP-related complications are still threatening maternal and fetal life and health. The prognosis of HDP is associated with the severity of disease process. In general, the more severe the disease is, the poorer prognosis is. Despite a massive research effort, there was also lack of efficient therapeutic methods in clinic at present. For the unpredictable characteristic and potential poor prognosis, symptomatic treatment to relieve clinical symptoms and timely termination of pregnancy were the main treatment measures, which can effectively increase curative rate and decrease complication rate and mortality.

Over the past few decades, relevant literatures about HDP have been focused on a small sample of individual hospitals or a single complication, lacking of large-scale samples with multi-variables research. Such previous works were not sufficient to provide detailed information for the current situation of HDP. Accordingly, our study was designed to perform a comprehensive retrospective survey with a large-scale sample collected at 38 hospitals across 14 provinces and regions in China mainland. The aims of this survey were to analyze the prevalence and possible risk factors of HDP, to investigate the effect of HDP and HDP related complications on maternal and perinatal outcomes.

## Methods

### Study Population

**Figure 1:**
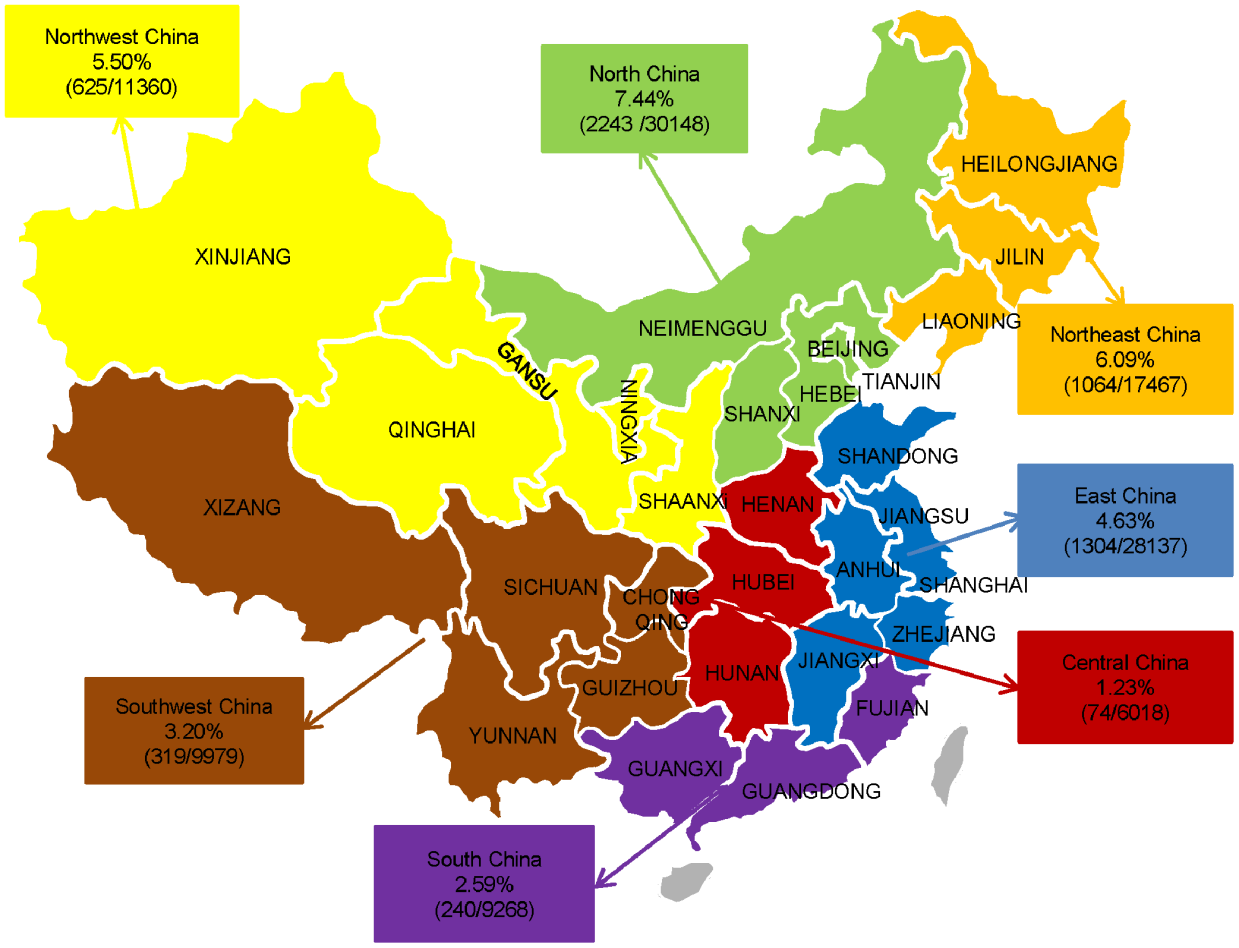
The prevalence of HDP in China during 2011.

Thirty-eight general or maternity hospitals were randomly selected from 14 provinces and regions over the country (except Hong Kong and Macau), covering the secondary and tertiary hospitals. As shown in Figure 1, these 14 provinces and regions located in seven territories of mainland China, including North China, Northeast China, Northwest China, East China, Southwest China, South China and Central China. In mainland China, a level 3A hospital is the highest level of care and a level 2A gives middle level of care. A total of 112,386 pregnant women, who gave birth between January 1^st^ 2011 and December 31^st^ 2011, were investigated. This study was approved by the Ethics Committees of medical institutions involved and conformed to the guidelines of the Helsinki agreement and its amendments. The National Research Ethics Service had previously approved the anonymous use of these data for research purposes, and hence, individual informed consent was not required. Clinical information stored in an electronic format was collected retrospectively from all the patients by reviewing their clinical medical recording files, not by face-to face or direct telephone calls.

A research database was retrospectively constructed with information regarding the maternal demographic data, reproductive history, family history, characteristics of pregnancy and delivery in the period between the first antenatal visit and postpartum. All information was collected on the basis of standardized antenatal, obstetric and neonatal records.

### Selection Criteria

The diagnosis of HDP was made based on its occurrence in the period between antepartum and postpartum. According to the latest version of classification system by the National High Blood Pressure Education Program (NHBPEP) Working Group, HDP was categorized into six subtypes, which include: gestational hypertension (GH), mild preeclampsia, severe preeclampsia, eclampsia, preeclampsia superimposed on chronic hypertension (PSCH) and chronic hypertension in pregnancy (CHP) [4]. GH is defined as a systolic blood pressure (SBP) of ≥140 mmHg and/or diastolic blood pressure (DBP) of ≥90 mmHg without proteinuria, which developed after 20 weeks of gestation and returned to normal within 12 weeks of postpartum. Mild preeclampsia is defined by the minimum criteria of blood pressure ≥140/90 mmHg after 20 weeks of gestation and proteinuria of ≥300 mg in a 24-hour urine specimen or 1+ in two random urine samples collected at least 4 hours apart. Severe preeclampsia is diagnosed if there are more severe elevations of blood pressure or evidence of other end-organ dysfunction. Preeclampsia is considered severe if one or more of the following criteria is present as follows (ACOG, 2002) [5]: (1) systolic blood pressure (SBP) of ≥160 mmHg and/or diastolic blood pressure (DBP) of ≥110 mmHg; (2)Proteinuria of ≥5 g in 24-hour urine or ≥3+ in two random urine samples collected at least 4 hours apart; (3) Oliguria of less than 500 ml in 24 hours; (4) Cerebral or visual disturbances; (5) Pulmonary edema or cyanosis; (6) Epigastric or right upper-quadrant pain; (7) Impaired liver function; (8) Thrombocytopenia(platelet counts <100,000/mm3); (9) Fetal growth restriction.

Eclampsia is the occurrence of a seizure in women in association with preeclampsia, in the absence of any other cause for seizures. PSCH is defined as the new establishment of proteinuria of ≥300 mg in 24 h urine in women of chronic hypertension which before 20 weeks of gestation but without proteinuria, or women of chronic hypertension with proteinuria before 20 weeks of gestation but appeared in urine protein or a sudden increase in blood pressure or platelet counts <100,000/mm3. CHP is defined as blood pressure of ≥140/90 mmHg before pregnancy or diagnosed prior to 20 weeks of gestation, or developed hypertension after 20 weeks of gestation and continued for 12 weeks of postpartum. It is very important to differentiate these 6 subtypes of HDP in determining prognosis and management [6].

The following factors were used as variables associated with risk of HDP in the present study: (1) body mass index (BMI) is calculated by weight in kilograms divided by (height in meters squared), in which BMI of 24.0–27.9 kg/m^2^ is defined as overweight and ≥28 kg/m^2^ as obese in Chinese population(in this study, we study the pre-pregnancy BMI) [7]; (2) preterm birth is defined as that occurring after 20 weeks and before 37 completed weeks of gestation internationally. This study used the Chinese standard (between 28 and 37 weeks of gestation); (3) neonatal asphyxia is defined as a hypoxia status, in which the baby only had a heartbeat but failed to breath at all or failed to establish regular breathing with a minute after fetal delivery (an Apgar score of less than 7 at 5 minutes after birth). (4) Fetuses and neonates who were born dead, or died in the first 28 days after delivery, were considered as perinatal dealth. (5) Neonates who were born with birth weights lower than 2500 g were considered as low birth weight (LBW) [6]. (6) Maternal complications are some conditions secondary to HDP, such as placental abruption, heart failure, renal failure, DIC, HELLP syndrome and cerebral vascular accident. We use the International Classification of Diseases (ICD-10) codes according to the hospital medical recording files to identity women who had complications.

We first examined the maternal characteristics of study subjects with HDP and then constructed a research database with the following information: (1) general information including maternal age (stratified as under the age of 20, 20–24, 25–29, 30–34, 35–39, 40 years and older), pre-pregnancy BMI, basal blood pressure that was measured at the first antenatal visit in first trimester or not (including basal SBP, basal DBP), ABO blood types, smoking, drinking and education; (2) reproductive history of pregnancies including gravidity, parity, number of abortions, history of stillbirths and HDP; (3) family history of hypertension and type-2 diabetes mellitus (T2DM) in parents of pregnant women; (4) clinical data including anemia, gestational DM (GDM) or T2DM, onset season, paternal age (stratified as under the age of 35, 35 years and older),singleton or twin pregnancies, fertilization method, modes of deliveries, sex of infants, birth weight, Apgar score, live birth and stillbirths; (5) maternal complications as mentioned above.

### Statistical Analyses

All data were inputted into computer for statistical analysis. Statistical analyses were performed using Microsoft Excel 2010 and SPSS version18.0. U test or χ2 test was used to compare frequencies among the categorical data. The risk factors identified were encoded as independent variables (Table S1 in File S1). Logistic regression models were used to identify the potential risk factors that might be associated with HDP, with calculation of odds ratios (OR) with 95% confidence intervals (CI). The model parameters were estimated by a maximum likelihood ratio test, which maximizes the likelihood of the observed data through several detections. Using univariate regression analysis to conduct the preliminary screening, a total of 23 independent variables were identified and selected for analysis, some of which were confirmed as the major factors associated with HDP. Multivariable logistic regression analysis was performed to determine the independent risk factors for the development of HDP using selected parameters with a P-value of <0.05 that was obtained from univariate regression analysis. When the occurrence of outcome was low (<10%), we assumed that the OR was equal to the relative risk. The risk factors with an OR value of >1.5 were defined as a major risk factor for HDP. All statistical tests and the value were two-sided, and the limit of statistical significance was P<0.05.

### Quality Assurance

All the subjects were interviewed with a questionnaire by specially trained professional person in a standardized way. Building an investigating collaborative group in collaboration with the person in charge of the assisted hospitals, to check out each questionnaire, and carry out random sampling and result to determine. Data were collected and recorded by specially trained medical staff (obstetrics and gynecology doctors and students) and randomly cross-checked by designated staff (high qualification doctors of obstetrics and gynecology). Statistical analysis was under the guidance and supervision of a professional statistician, so as to ensure the accuracy of the analysis results.

## Results

Of the 112,386 pregnant women whose information was sampled, of which 5869 (5.22%) were identified with HDP, while the rest of 106517 with normotensive as the control group. Among them, 91171 were primipara and 21215 were multipara (Table 1). Among the patients with HDP, 78.99% were 20–34 years old (29.93±6.38 years old), 75.99% experienced their first parity, and in 90.69%, hypertension were diagnosed in the third trimester of pregnancy or 12 weeks after delivery (34.84±5.46 weeks).

**Table 1:** Demographic characteristics of the 112,386 pregnant women in the study.

		All subjects	Constituent Ratio %	Subjects with HDP		
				N	%	Constituent Ratio %
**Age (years)**	<20	1528	1.36	78	5.10	1.33
	20–24	24260	21.59	1064	4.39	18.13
	25–29	46882	41.72	2028	4.33	34.55
	30–34	27628	24.58	1544	5.59	26.31
	35–39	9253	8.23	872	9.42	14.86
	≥40	1979	1.76	252	12.73	4.29
	Unknown	856	0.76	31	3.62	0.53
**Pre-pregnancy BMI (kg/m2)**	<24.0	53957	48.01	2165	3.81	36.89
	24.0∼	9646	8.58	824	8.54	14.04
	≥28.0	2367	2.11	400	16.90	6.81
	Unknown	46416	41.30	2480	5.34	42.26
**Gravidity**	1	56828	50.57	2656	4.67	45.25
	2	30721	27.34	1603	5.22	27.31
	≥3	24837	22.10	1610	6.48	27.43
**Parity**	1	91171	81.12	4460	4.89	75.99
	2	19075	16.97	1209	6.34	20.60
	3	1923	1.71	177	9.20	3.02
	≥4	217	0.19	23	10.60	0.39
**Abortion**	0	69593	61.92	3328	4.78	56.70
	1	26509	23.59	1506	5.68	25.66
	2	11137	9.91	694	6.23	11.82
	≥3	5147	4.58	341	6.63	5.81
**Education level**	Above university	55586	49.46	2715	4.88	46.26
	Secondary school	30000	26.69	1579	5.26	26.90
	Primary school	22531	20.05	1301	5.77	22.17
	Illiteracy	260	0.23	23	8.85	0.39
	Unknown	4009	3.57	251	6.26	4.28
**Pregnancy**	Singleton	110385	98.22	5543	5.02	94.44
	Twin	2001	1.78	326	16.29	5.56

### The Prevalence of HDP in China

A total of 112,386 pregnant women were recorded within the study period and their demographic characteristics were given in Table 1. Among the study population, 5,869 developed HDP (5.22% of all deliveries during 2011), of whom 2,016 (31.40%) were diagnosed as having GH, 888 (15.13%) as having mild preeclampsia, 2345 (39.96%) as having severe preeclampsia, 52 (0.89%) as having eclampsia, 216 (3.68%) as having PSCH and 352 (6.00%) as having CHP. Of the 112,386 pregnant women whose information was sampled, of which 5869 (5.22%) were identified with HDP, while the rest of 106517 with normotensive as the control group.(Figure 1).

### The Possible Risk Factors for HDP

For HDP as dependent variable Y (Yes = 1, No = 0) and various factors as independent variables, logistic regression models were constructed to analysis these interactions. (Table S1 in File S1). A total of 23 independent variables were selected for analysis, 18 of which were confirmed to be possible risk factors by univariate regression analysis of individual variables (Table 2). The factors which had an effect size of OR >1.5 (P<0.001) included twin pregnancy (OR = 3.68, 95%CI 3.26∼4.16), alcohol consumption (OR = 1.75, 95%CI 1.45∼2.11), family histories of hypertension (OR = 3.17, 95%CI 2. 71∼3.71) and T2DM (OR = 2.67, 95%CI 2. 07∼3.44), high basal SBP(≥70 mmHg) (OR = 2.81, 95%CI 2.66∼2.97), alcohol consumption (OR = 1.75, 95%CI 1.45∼2.11), high basal DBP (≥120 mmHg)(OR = 1.67, 95%CI 1.59∼1.77) and GDM (OR = 2.48, 95%CI 2. 28∼2.69).

**Table 2:** Univariate regression analysis of possible risk factors for HDP.

Related factors	B	S.E.	Wald	df	P	OR	95% CI
**Maternal age**	0.055	0.003	436.36	1	<0.001	1.06	1.05∼1.06
**Gravidity**	0.169	0.016	108.13	1	<0.001	1.18	1.15∼1.22
**Parity**	0.296	0.025	136.30	1	<0.001	1.34	1.28∼1.41
**History of abortion**	0.116	0.013	78.34	1	<0.001	1.12	1.09∼1.15
**Twin pregnancy**	1.303	0.062	440.65	1	<0.001	3.68	3.26∼4.16
**Education level**	0.062	0.016	15.39	1	<0.001	1.06	1.03∼1.10
**Alcohol consumption**	0.560	0.096	33.85	1	<0.001	1.75	1.45∼2.11
**Family history of hypertension**	1.153	0.080	208.46	1	<0.001	3.17	2.71∼3.71
**Family history of T2DM**	0.981	0.130	57.37	1	<0.001	2.67	2.07∼3.44
**Pre-pregnancy BMI**	0.155	0.005	1049.49	1	<0.001	1.17	1.16∼1.18
**Basal SBP**	1.033	0.027	1418.39	1	<0.001	2.81	2.66∼2.97
**Basal DBP**	0.515	0.027	363.44	1	<0.001	1.67	1.59∼1.77
**Fertilization method**	0.711	0.081	76.868	1	<0.001	2.04	1.75∼2.39
**ABO blood type**	0.040	0.011	13.17	1	<0.001	1.04	1.02∼1.06
**GDM**	0.907	0.041	478.78	1	<0.001	2.48	2.28∼2.69
**Anemia**	0.136	0.052	6.73	1	0.009	1.15	1.03∼1.27
**Onset season**	−0.063	0.031	4.165	1	0.041	0.94	0.89∼0.10
**Paternal age**	0.492	0.034	204.202	1	<0.001	1.64	1.53∼1.75

Although co-linearity diagnostics with these 18 HDP-underlying factors failed to show co-linearity between these variables (Table S2 in File S1), multivariate logistic regression analysis identified some additional risk factors with an OR of >1.5 (Table S3 in File S1). Pregnant women aged 25–29 years had the lowest risk of HDP (the prevalence of 4.33%) compared with those aged 35–39 years (9.42%, adjusted OR = 1.84, 95%CI 1.62∼2.09, P<0.001) and those aged 40 years and older(12.73%, adjusted OR = 2.39, 95%CI 1.86∼2.91, P<0.001). Compared with women who had a normal pre-pregnancy BMI, the risk of HDP was 1.8 times higher in those who were overweight (adjusted OR = 1.79, 95%CI 1.63∼1.96, P<0.001) and 3.1 times higher in those obese (adjusted OR = 3.11, 95%CI 2.71∼3.56, P<0.001). Multiparous women had a lower risk of HDP than those who were primipara (adjusted OR = 0.66, 95%CI 0.58∼0.76, P<0.001). Both parity and history of abortion appeared to make further contribution to the development of HDP: with women who had >3 parities being more likely to develop HDP than those who had one previous childbirth (adjusted OR = 2.05, 95%CI 1.18∼3.56, P<0.001), and women who had history of abortion was also at a higher risk of HDP (Table S3 in File S1). The prevalence of pre-term birth, placental abruption, postpartum hemorrhage and caesarean delivery rate were significantly higher in women with HDP than those without HDP (Table 3). It was also found that there was also a significant difference between the six subtypes of HDP. The incidence rates of adverse pregnancy outcomes in eclampsia, severe preeclampsia and PSCH group were significant higher than that in patients with GH and normal pregnancy group (Table S4 in File S1).

**Table 3:** Differences in pregnancy outcomes between women with and without HDP.

Group	N	Preterm Birth (%)	PlacentalAbruption (%)	PostpartumHemorrhage (%)	CesareanSection (%)
**GH**	2016	227(11.26)	16(0.79)	143(7.09)	1327(65.82)
**Mild Preeclampsia**	888	128(14.41)	19(2.14)	58(6.53)	695(78.27)
**Severe Preeclampsia**	2345	1166(49.72)	129(5.50)	132(5.63)	2023(86.27)
**Eclampsia**	52	37 (71.15)	6(11.54)	1(1.92)	49(94.23)
**PSCH**	216	113(52.31)	14(6.48)	13(6.02)	176(81.48)
**CHP**	352	52(14.77)	4(1.14)	31(8.81)	246(69.89)
**With HDP**	5869	1723(29.36)	188(3.20)	378(6.44)	4516(76.95)
**Without HDP**	106517	7226(6.78)	452(0.42)	3821(3.59)	56834(53.36)
**Total**	112386	8949(7.96)	640(0.57)	4199(3.74)	61350(54.59)
**X2**		3867.7	758.6	125.9	1248.698
**P**		<0.001	<0.001	<0.001	<0.001

### The HDP-related Complications

As shown in Table 4, the prevalence of low birth weight(LBW, <2500 g), neonatal asphyxia and perinatal deaths were significantly higher in women with HDP than in those without HDP (P<0.001). Among 5,869 women with HDP, 4,516 (76.95%) had cesarean sections (CS). The CS rate in women with HDP was significantly higher than those without HDP (53.36%), with an OR of 2.92 and 95% CI 2.74∼3.10 (X^2^ = 1248.7, P<0.001).

**Table 4:** Differences in perinatal outcomes between women with and without HDP.

Group	N	LBW (%)	Neonatal Asphyxia (%)	Perinatal Death (%)
**GH**	2091	214(10.23)	102(4.88)	44(2.10)
**Mild Preeclampsia**	949	159(16.75)	58(6.11)	11(1.16)
**Severe Preeclampsia**	2522	1134(44.96)	479(18.99)	206(8.17)
**Eclampsia**	53	40(75.47)	24(45.28)	5(9.43)
**PSCH**	222	93(41.89)	66(29.73)	31(13.96)
**CHP**	358	40(11.17)	31(8.66)	17(4.75)
**With HDP**	6195	1697(27.39)	760(12.27)	314(5.07)
**Without HDP**	108192	6167(5.70)	3689(3.41)	1456(1.35)
**Total**	114387	7864(6.87)	4449(3.89)	1770(1.55)
**X2**		4306.9	1230.0	533.1
**P**		<0.001	<0.001	<0.001

The prevalence of severe pregnancy complications varied among women with HDP: placental abruption showed the highest (3.20%) and DIC showed the lowest(0.12%) (Table 5).

**Table 5:** Prevalence of HDP-related complications.

HDP	N	Placental abruption(%)	HELLP Syndrome(%)	Heart failure (%)	Renal failure (%)	Cerebrovascular accident (%)	Pulmonary edema (%)	DIC (%)
**GH**	2016	16(0.79)	3(0.15)	3(0.15)	1(0.05)	1(0.05)	1(0.05)	1(0.05)
**Mild preeclampsia**	888	19(2.14)	5(0.56)	3(0.34)	0	0	1(0.11)	1(0.11)
**Severe preeclampsia**	2345	129(5.50)	79(3.37)	24(1.02)	12(0.51)	13(0.55)	5(0.21)	5(0.21)
**Eclampsia**	52	6(11.54)	5(9.62)	0	0	5(9.62)	0	0
**PSCH**	216	14(6.48)	4(1.85)	0	3(1.39)	1(0.46)	0	0
**CHP**	352	4(1.14)	0	0	0	0	0	0
**Total**	**5869**	**188(3.20)**	**96(1.64)**	**30(0.51)**	**16(0.27)**	**20(0.34)**	**7(0.12)**	**7(0.12)**

Of 5,869 women with HDP, 5 died and the causes of maternal death were functional failures of the main organs (heart, lung and kidney, 3 cases), severe pulmonary hypertension associated with HELLP syndrome (1 case), the rest cause of death was unknown. Of these 5 maternal deaths, 3 occurred in patients with severe preeclampsia. By contrast, among 106,517 women without HDP, 8 died and the main causes of maternal deaths were amniotic fluid embolism, DIC and heart failure.

## Discussion

The present study revealed that the 5.22% prevalence of HDP in the Chinese female population in 2011 was lower than that reported in other populations, such as African Americans (6.4%), and Brazilians (7.5%) [8]–[9]. This variation between populations may be attributed to the differences in ethnic background, age distribution, socio-economic status, and the number of times of gravidity and parity. It is worth noting that the 1988 survey on HDP reported a prevalence of 9.4% in China [10], while this 2011 nationwide study suggests that the prevalence of HDP has come down in recent years in the Chinese population. It may be attributed to the fact that sampling was performed from different geographical areas in China. We found that, there is a large difference in the prevalence of HDP between geographical regions across the country (Figure 1). This may be associated with the rapid development of the economy in China, which could facilitate an improvement in living standards, education level, medication and health care, such as the improvement of HDP prediction technology and precautionary measures. While imbalanced development of regional economy and socio-demographic differences are the most likely reasons in China, different climate and weather may also contribute to an increased risk of HDP. Some data tend to suggest that eclampsia is associated with cooler temperatures or winter or with increased humidity or rainfall [11]–[12]. The exact mechanism is beyond the scope of our study. Further investigation is needed to confirm whether a low temperature-induced vasoconstriction may trigger an onset of HDP, leading to the highest prevalence of the condition in this region. The Northern China has a colder climate and longer winter time as compared to other regions. It still needs further study.

Apart from the environmental factors, individual background in genetics and medical history are factors that influence the development of HDP. The risk of HDP was 1.75 times in alcohol consumers comparing with no alcohol consumers. Epidemiological studies have demonstrated a close association between alcohol use and an increased risk of hypertension [13]–[15], but few studies have directly addressed the role of drinking pattern. In our study, hypertension has no obvious relationship with cigarette smoking. Paradoxically, Studies have shown that smoking during pregnancy has been associated with a reduced risk of preeclampsia [16]. For example, smoking during pregnancy reduces the risk of preeclampsia by up to 50% with a dose-response pattern [17]. Numerous studies have shown that twin pregnancy is an important risk factor for HDP. Our study suggested that 16.29% of 2001 women with a twin pregnancy developed HDP as compared with 5.02% of 110,385 women with a single pregnancy (Table 1). This result strongly supports previous reports indicating that the risk of HDP in women with a twin pregnancy was 2 or 3 times higher than those with a single pregnancy [18]–[20]. Numbers of studies indicate that the prevalence of HDP increases with maternal age [19], [21]–[22]. For an example, the risk of preeclampsia has been shown to increase by 30% with every year above the age of 34 years [23]–[24]. In the current study, we have shown similar results: compared with women aged 20–24 years, the risk of HDP was 1.8 times higher in those aged 35–39 years and 2.4 times higher in those aged 40 years and older (Table 1 and Table S3 in File S1). It has been frequently reported that HDP commonly occurs in the first delivery [25]–[26].

There is a close relationship between HDP and pre-pregnancy BMI, including the increased risk of HDP in women with obesity during pregnancy [19], [27]–[29]. Some studies suggest that each increase of 5 to 7 kg/m^2^ in BMI doubles the risk of developing preeclampsia [30]–[31]. Obese women were at a higher risk of preeclampsia compared to those with a normal BMI [32]. Our work confirms the association of body weight with risk of HDP: obese women had the highest risk of HDP (Table 1 and Table S3 in File S1). In recent years, attention has been paid to the relationship between the morbidity in HDP and the levels of maternal education. Convincing evidence suggests that women with a low level of education are more likely to develop HDP than those who have received a higher level of education [33]–[34]. The present study provides weak evidence in support of the impact of education levels on the risk of HDP (Table S3 in File S1). Epidemiological data indicate that HDP shows a trend towards familial aggregation [35]–[36].The prevalence of HDP in women who had two or more family members with HDP was 2 to 3 times higher than that the general female population [19], [37]. High blood pressure was an independent risk factor for preeclampsia [38]. SBP at first antenatal visit was positively associated with the risk of preeclampsia; Women with SBP of ≥130 mmHg had a relative risk of 3.6 compared to those with pre-pregnancy SBP of <110 mmHg [39]. In a retrospective cohort study, Stamilio et al found that a mean arterial pressure of >90 mmHg measured in the first prenatal visit was associated with a high risk of severe preeclampsia [40]. It has been suggested that blood pressure is an indicator of the degree of HDP [41]–[43]. Our work also suggests that GDM is a risk factor for HDP. Similar findings have been reported in several previous studies [44]–[45].

### Conclusions

The pathogenesis mechanism behind of HDP remains to be fully understood. It is difficult to make effective diagnosis and treatment method that can be directed towards the pathogenesis of the condition. The present report describes a large cohort retrospective survey on HDP and has identified a number of risk factors for the condition in the Chinese population. To our knowledge, this is the largest epidemiological survey reported to date and this work has provided comprehensive information about the risk factors involved in HDP among the Chinese population. Identification of these risk factors for HDP would be useful for early diagnosis of HDP in a particular patient group that requires clinical monitoring and appropriate treatment. In future studies, it is critical to find effective interventions and preventions of HDP which are particularly important to reduce maternal and perinatal complications, and ensure both pregnant women and infants to be healthy and safe.

### Strengths and Limitations

As a multicenter clinical epidemiological research, we had the large number of 112386 patients came from thirty-eight hospitals of 14 provinces and regions over the Chinese country, while most other studies present a smaller number of patients derived from one hospital or from a local area. As a retrospective study, part of the clinical data was not completed, which undetected and deviations can exist. Some data such as income, personality, diet, drug abuse, etc. cannot be obtained from cases, so it can not represent all of the risk factors.

## Supporting Information

File S1 **Supporting tables. Table S1,** Assignment of potential risk factors for HDP. **Table S2,** Collinearity diagnostics of independent variables. **Table S3,** Multivariate logistic analysis of 18 risk factors for HDP. **Table S4,** Differences in outcomes between HDP.
(DOC)
