# Supplementary material for: The 2011 Survey on Hypertensive Disorders of Pregnancy (HDP) in China: Prevalence, Risk Factors, Complications, Pregnancy and Perinatal Outcomes
Source: PLoS One. 2014 Jun 17;9(6):e100180. doi: 10.1371/journal.pone.0100180 (PMC4061123; doi:10.1371/journal.pone.0100180)
Supplement: File S1 — Supporting tables. Table S1, Assignment of potential risk factors for HDP. Table S2, Collinearity diagnostics of independent variables. Table S3, Multivariate logistic analysis of 18 risk factors for HDP. Table S4, Differences in outcomes between HDP. (DOC) [file pone.0100180.s001.doc]

1. Assignment of potential risk factors for HDP

| **Factors** | **Variables** | **Assignment Instructions** |
| --- | --- | --- |
| **HDP** | Y | No=0, Yes=1 |
| **Maternal age** | X1 | 20-24=0,＜20=1, 25-29=2, 30-34=3, 35-39=4, ≥40=5, Unknown =6 |
| **Gravidity** | X2 | First=1, Second=2, Third and above =3 |
| **Parity** | X3 | First=0, Second=1, Third=2, forth=3 |
| **History of abortion** | X4 | No=0, 1 =1, 2 =2, ≥3=3 |
| **Multiple births** | X5 | No =0, Yes =1 |
| **History of fetal deaths** | X6 | No =0, Yes =1 |
| **History of stillbirths** | X7 | No =0, Yes =1 |
| **Educational level** | X8 | Above university=1, More than secondary and high school=2, Under junior high school=3, Illiteracy=4，Unknown =5 |
| **Smoking** | X9 | No =0, Yes =1 |
| **Alcohol consumption** | X10 | No =0, Yes =1 |
| **Family history of**  **hypertension** | X11 | No =0, Yes =1 |
| **Family history of T2DM** | X12 | No =0, Yes =1 |
| **Pre-pregnancy BMI** | X13 | ＜24=0, 24-27.9=1, ≥28=2, Unknown=3 |
| **Systolic blood pressure** | X14 | ＜120=0, ≥120=1 |
| **Diastolic blood pressure** | X15 | ＜70=0, ≥70=1 |
| **Way of pregnancy** | X16 | Nature=1, IVF-ET=2, Others=3 |
| **Sex of infants** | X17 | Boy=0, Girl=1 |
| **ABO blood type** | X18 | A =1, B =2, AB =3, O=4 |
| **Rh blood type** | X19 | positive =1, negative=2 |
| **GDM** | X20 | No =0, Yes =1 |
| **Anemia** | X21 | No =0, Yes =1 |
| **Season of onset** | X22 | Spring=1, Summer=2, Fall=3, Winter=4 |
| **Age of husband** | X23 | ＜35=0, ≥35=1 |

1. Collinearity diagnostics of independent variables

| **Related factors** | **Variables** | **Collinearity statistics** | |
| --- | --- | --- | --- |
|  |  | **Variable tolerance** | **Variance inflation factor** |
| **Maternal age** | X1 | 0.592 | 1.690 |
| **Gravidity** | X2 | 0.187 | 5.335 |
| **Parity** | X3 | 0.508 | 1.970 |
| **Abortion number** | X4 | 0.247 | 4.052 |
| **Multiple pregnancy** | X5 | 0.950 | 1.053 |
| **Education level** | X8 | 0.871 | 1.148 |
| **Alcohol consumption** | X10 | 0.993 | 1.007 |
| **Family history of Hypertension** | X11 | 0.898 | 1.114 |
| **Family history of T2DM** | X12 | 0.943 | 1.060 |
| **Pre-pregnancy BMI** | X13 | 0.733 | 1.364 |
| **Systolic blood pressure** | X14 | 0.769 | 1.300 |
| **Diastolic blood pressure** | X15 | 0.615 | 1.625 |
| **Way of pregnancy** | X16 | 0.944 | 1.059 |
| **ABO blood type** | X18 | 0.998 | 1.002 |
| **GDM** | X20 | 0.973 | 1.028 |
| **Anemia** | X21 | 0.989 | 1.011 |
| **Season of onset** | X22 | 0.913 | 1.095 |
| **Age of husband** | X23 | 0.653 | 1.530 |

1. Multivariate logistic analysis of 18 risk factors for HDP

| **Risk factors** | | | | **B** | **S.E.** | **Wald** | **P** | **OR** | **95% CI** |
| --- | --- | --- | --- | --- | --- | --- | --- | --- | --- |
| **Maternal age** | | **20-24** | |  |  | 179.48 | <0.001 |  |  |
|  |  | **＜20** | | 0.132 | 0.154 | 0.73 | 0.392 | 1.14 | 0.84~1.54 |
|  |  | **25-29** | | -0.018 | 0.049 | 0.14 | 0.711 | 0.98 | 0.89~1.08 |
|  |  | **30-34** | | 0.168 | 0.054 | 9.69 | 0.002 | 1.18 | 1.06~1.32 |
|  |  | **35-39** | | 0.609 | 0.066 | 86.05 | <0.001 | 1.84 | 1.62~2.09 |
|  |  | **≥40** | | 0.870 | 0.101 | 73.66 | <0.001 | 2.39 | 1.96~2.91 |
| **Gravidity** | | **1** | |  |  | 37.24 | <0.001 |  |  |
|  |  | **2** | | -0.416 | 0.069 | 36.53 | <0.001 | 0.66 | 0.58~0.76 |
|  |  | **≥3** | | -0.565 | 0.112 | 25.62 | <0.001 | 0.57 | 0.46~0.71 |
| **Parity** | | **1** | |  |  | 28.90 | <0.001 |  |  |
|  |  | **2** | | 0.233 | 0.061 | 14.47 | <0.001 | 1.26 | 1.12~1.42 |
|  |  | **3** | | 0.570 | 0.122 | 22.02 | <0.001 | 1.77 | 1.39~2.25 |
|  |  | **≥4** | | 0.718 | 0.281 | 6.53 | 0.011 | 2.05 | 1.18~3.56 |
| **History of abortion** | | **0** | |  |  | 41.27 | <0.001 |  |  |
|  |  | **1** | | 0.438 | 0.070 | 39.49 | <0.001 | 1.55 | 1.35~1.78 |
|  |  | **2** | | 0.525 | 0.105 | 25.15 | <0.001 | 1.69 | 1.38~2.08 |
|  |  | **≥3** | | 0.405 | 0.117 | 11.90 | <0.001 | 1.50 | 1.19~1.89 |
| **Twin pregnancy** | | | | 1.168 | 0.083 | 199.97 | <0.001 | 3.22 | 2.76~3.78 |
| **Education**  **level** | **Above university** | | |  |  | 10.44 | 0.034 |  |  |
|  | **secondary school** | | | -0.002 | 0.041 | 0.01 | 0.958 | 1.00 | 0.92~1.08 |
|  | **Primary school** | | | 0.122 | 0.048 | 6.42 | 0.011 | 1.13 | 1.03~1.24 |
|  | **Illiteracy** | | | 0.427 | 0.292 | 2.14 | 0.143 | 1.53 | 0.87~2.71 |
| **Alcohol consumption** | | | | 0.625 | 0.108 | 33.20 | <0.001 | 1.87 | 1.51~2.31 |
| **Family history of hypertension** | | | | 1.042 | 0.091 | 130.21 | <0.001 | 2.84 | 2.37~3.39 |
| **Family history of T2DM** | | | | 0.469 | 0.151 | 9.64 | 0.002 | 1.60 | 1.19~2.15 |
| **Pre-pregnancy BMI** | | | **＜24** |  |  | 604.46 | <0.001 |  |  |
|  |  |  | **24-27.9** | 0.581 | 0.048 | 146.89 | <0.001 | 1.79 | 1.63~1.96 |
|  |  |  | **≥28** | 1.134 | 0.070 | 265.43 | <0.001 | 3.11 | 2.71~3.56 |
| **SDP** | | | | 1.112 | 0.042 | 699.40 | <0.001 | 3.04 | 2.80~3.30 |
| **DBP** | | | | 0.575 | 0.050 | 131.91 | <0.001 | 1.78 | 1.61~1.96 |
| **ABO blood type** | | | **A** |  |  | 21.51 | <0.001 |  |  |
|  |  |  | **B** | 0.166 | 0.042 | 15.56 | <0.001 | 1.18 | 1.09~1.28 |
|  |  |  | **AB** | 0.169 | 0.061 | 7.553 | 0.006 | 1.18 | 1.05~1.34 |
|  |  |  | **O** | 0.096 | 0.043 | 5.01 | 0.025 | 1.10 | 1.01~1.20 |
| **GDM** | | | | 0.454 | 0.056 | 65.34 | <0.001 | 1.57 | 1.41~1.76 |

1. Differences in outcomes between HDP

| **Group** | **Case** | **Preterm**  **birth** | **Placental**  **abruption** | **Postpartum hemorrhage** | **Caesarean section** |
| --- | --- | --- | --- | --- | --- |
| **GH** | 2016 | 227(11.26%) | 16(0.79%) | 143(7.09%) | 1327(65.82%) |
| **Mild preeclampsia** | 888 | 128(14.41%) | 19(2.14%) | 58(6.53%) | 695(78.27%) |
| **Severe preeclampsia** | 2345 | 1166(49.72%) | 129(5.50%) | 132(5.63%) | 2023(86.27%) |
| **Eclampsia** | 52 | 37 (71.15%) | 6(11.54%) | 1(1.92%) | 49(94.23%) |
| **PSCH** | 216 | 113(52.31%) | 14(6.48%) | 13(6.02%) | 176(81.48%) |
| **CHP** | 352 | 52(14.77%) | 4(1.14%) | 31(8.81%) | 246(69.89%) |
